# H_2_O_2_ Functions as a Downstream Signal of IAA to Mediate H_2_S-Induced Chilling Tolerance in Cucumber

**DOI:** 10.3390/ijms222312910

**Published:** 2021-11-29

**Authors:** Xiaowei Zhang, Yanyan Zhang, Chenxiao Xu, Kun Liu, Huangai Bi, Xizhen Ai

**Affiliations:** State Key Laboratory of Crop Biology, Key Laboratory of Crop Biology and Genetic Improvement of Horticultural Crops in Huanghuai Region, College of Horticulture Science and Engineering, Shandong Agricultural University, Tai’an 271018, China; 2019010077@sdau.edu.cn (X.Z.); zyy725619@163.com (Y.Z.); xcx18853812186@163.com (C.X.); ulking1223@163.com (K.L.); bhg163@163.com (H.B.)

**Keywords:** chilling stress, hydrogen sulfide, hydrogen peroxide, indole-3-acetic acid, signaling pathway

## Abstract

Hydrogen sulfide (H_2_S) plays a crucial role in regulating chilling tolerance. However, the role of hydrogen peroxide (H_2_O_2_) and auxin in H_2_S-induced signal transduction in the chilling stress response of plants was unclear. In this study, 1.0 mM exogenous H_2_O_2_ and 75 μM indole-3-acetic acid (IAA) significantly improved the chilling tolerance of cucumber seedlings, as demonstrated by the mild plant chilling injury symptoms, lower chilling injury index (CI), electrolyte leakage (EL), and malondialdehyde content (MDA) as well as higher levels of photosynthesis and cold-responsive genes under chilling stress. IAA-induced chilling tolerance was weakened by N, N′-dimethylthiourea (DMTU, a scavenger of H_2_O_2_), but the polar transport inhibitor of IAA (1-naphthylphthalamic acid, NPA) did not affect H_2_O_2_-induced mitigation of chilling stress. IAA significantly enhanced endogenous H_2_O_2_ synthesis, but H_2_O_2_ had minimal effects on endogenous IAA content in cucumber seedlings. In addition, the H_2_O_2_ scavenger DMTU, inhibitor of H_2_O_2_ synthesis (diphenyleneiodonium chloride, DPI), and IAA polar transport inhibitor NPA reduced H_2_S-induced chilling tolerance. Sodium hydrosulfide (NaHS) increased H_2_O_2_ and IAA levels, flavin monooxygenase (FMO) activity, and respiratory burst oxidase homolog (*RBOH1*) and FMO-like protein (*YUCCA2*) mRNA levels in cucumber seedlings. DMTU, DPI, and NPA diminished NaHS-induced H_2_O_2_ production, but DMTU and DPI did not affect IAA levels induced by NaHS during chilling stress. Taken together, the present data indicate that H_2_O_2_ as a downstream signal of IAA mediates H_2_S-induced chilling tolerance in cucumber seedlings.

## 1. Introduction

Cucumbers (*Cucumis sativus* L.) are typical light-loving and cold-sensitive plants, but they are mainly cultivated in solar greenhouses in northern China. When exposed to temperatures below 10 °C, cucumber plants generally suffer chilling injury (Ai et al.) [1]. Therefore, chilling is considered as a crucial limitation to growth and yield in cucumber production. Hydrogen sulfide (H_2_S) is a novel gaseous signaling molecule that plays an important role in regulating plant growth and development and defense responses to various abiotic stresses. Previous studies revealed that H_2_S upregulated the expression levels of mitogen-activated protein kinase (*MAPK*) and was involved in the upregulation of *MAPK* gene expression caused by cold stress [2]. The exogenous fumigation of H_2_S or application of sodium hydrosulfide (NaHS, the H_2_S donor), can relieve multiple abiotic stresses, such as chilling, heat, salinity, drought, hypoxia, and heavy metal toxicity [3]. We recently found that NaHS enhances the chilling tolerance of cucumber by scavenging reactive oxygen species (ROS), increasing CO_2_ assimilation, and upregulating the expression of cold-responsive genes [4]. Some signaling molecules, such as nitrogen monoxide (NO), Ca^2+^, abscisic acid (ABA), and indol-3-acetic acid (IAA) are involved in H_2_S-induced response to chilling stress in cucumber [4,5,6,7]. However, whether any other signaling molecules are involved in the process of H_2_S-induced chilling tolerance, the relationship between these signaling molecules remains unclear.

Studies over the last decades have indicated that endogenous hydrogen peroxide (H_2_O_2_) is induced in plants after exposure to abiotic stress, such as low or high temperature, heavy metals, water stress, etc. [8,9,10]. H_2_O_2_ interacts with other plant growth regulators, such as auxins, gibberellins, cytokinins, etc. (as signaling molecules) synergistically or antagonistically, and it mediates plant growth and development and responses to abiotic stresses [10]. Pasternak et al. [11] suggested that the variation of *PINOID* gene expression triggered by H_2_O_2_ influenced the polar transport of auxin and might alter auxin homeostasis. The application of H_2_O_2_ induced the formation of adventitious roots in *Linum usitatissimum* by regulating endogenous auxin levels [12]. Zhu et al. [13] demonstrated that ethylene and H_2_O_2_ play an important role in triggering brassinosteroid-induced salt tolerance in tomato plants. Our recent study suggests that H_2_O_2_ is involved in H_2_S-induced photoprotection in cucumber seedlings after exposure to chilling [14].

Auxin plays an essential role in the regulation of plant growth and development, but information about its role under chilling stress remains limited. Previous studies have revealed that changes in plant growth and development caused by cold stress are closely related to the intracellular auxin gradient, which is regulated by the polar deployment and intracellular trafficking of auxin transporters [15]. Recently, we found that IAA, a main auxin, could increase chilling tolerance by decreasing ROS accumulation, increasing the enzyme activities of photosynthesis and upregulating the expression of cold-responsive genes [4]. IAA also participates in the H_2_S-mediated response to chilling stress in cucumber, and it controls the H_2_O_2_ in the growing part of the root [16]. Therefore, we speculate that crosstalk may exist among H_2_O_2_, IAA, and H_2_S in response to chilling stress. To test this assumption, we investigated the effect of H_2_O_2_ and IAA on the ROS accumulation, photosynthesis, and relative expression of cold-responsive genes and the role of H_2_O_2_ and IAA in H_2_S-induced chilling tolerance in cucumber seedlings.

## 2. Results

### 2.1. H_2_O_2_ Is Involved in H_2_S-Induced Chilling Tolerance in Cucumber

**Figure 1:**
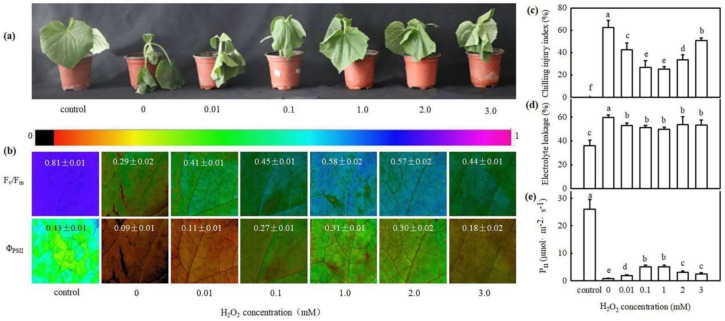
Effect of H_2_O_2_ on the chilling tolerance of cucumber seedlings. (**a**) Phenotype characterization of cucumber seedlings pretreated with H_2_O_2_ or deionized water under chilling stress (8/5 °C) for 48 h. Deionized water-treated seedlings before chilling stress were used as the control. The experiments were repeated three times with similar results. A typical picture is shown here. (**b**) Image of F_v_/F_m_ and Φ_PSII_ in seedlings before (control) and after chilling stress for 24 h. The false color code depicted at top of the image represents the degree of photoinhibition at PSII. (**c**) CI of seedlings before (control) and after chilling stress for 72 h. (**d**) EL of seedlings before (control) and after chilling stress for 48 h. (**e**) Pn of seedlings before (control) and after chilling stress for 24 h. Two-leaf stage cucumber seedlings were foliage sprayed with 0, 0.01, 0.1, 1.0, 2.0 and 3.0 mM H_2_O_2_ solution for 24 h and subsequently were exposed to 8/5 °C (day/night). The data represent the mean ± SD of three biological replicates. Different letters indicate significant differences (*p* < 0.05), according to Duncan’s new multiple range test.

To explore the effect of exogenous H_2_O_2_ on chilling tolerance in cucumber, we determined the maximum photochemical efficiency of PSII (*F*_v_/*F*_m_), actual photochemical efficiency of PSII (Φ_PSII_), chilling injury index (CI), electrolyte leakage (EL), and photosynthetic rate (P_n_) of cucumber seedlings, which were pretreated with different concentrations of H_2_O_2_ after exposure to 8/5 °C (day/night) for 24–72 h. As shown in Figure 1, H_2_O_2_ alleviated chilling injury symptoms in cucumber seedlings, and this alleviation effect was increased at low concentrations of H_2_O_2_ but was suppressed when the concentration exceeded 1.0 mM. The *F*_v_/*F*_m_, Φ_PSII_, and P_n_ of H_2_O_2_-treated seedlings were much higher, and the CI and EL were much lower than 0 mM H_2_O_2_ (H_2_O) treatments. These results reveal that H_2_O_2_ improves the chilling tolerance of cucumber seedlings, and its effect is concentration dependent. Thus, we use 1.0 mM H_2_O_2_ in further experiments.

**Figure 2:**
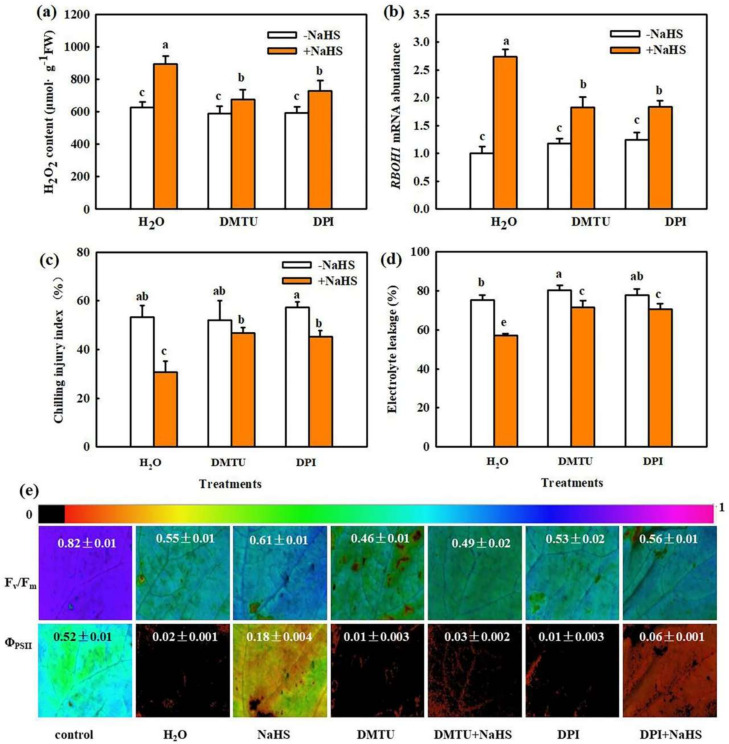
Effects of DMTU and DPI on H_2_S-induced H_2_O_2_ content, *RBOH1* mRNA abundance, and chilling tolerance in cucumber. (**a**) H_2_O_2_ content in seedlings before (control) and after chilling stress for 9 h; (**b**) mRNA abundance of *RBOH1* in seedlings before (control) and after chilling stress for 9 h. (**c**) CI of seedlings before (control) and after chilling stress for 72 h; (**d**) EL of seedlings before (control) and after chilling stress for 48 h; (**e**) Image of F_v_/F_m_ and Φ_PSII_ of seedlings before (control) and after chilling stress for 24 h. The false color code depicted at top of the image represents the degree of photoinhibition at PSII. Two-leaf stage cucumber seedlings were pretreated with DMTU, DPI, or deionized water and then sprayed with NaHS after 6 h. Twelve hours later, the seedlings were exposed to chilling stress. The data represent mean ± SD of three biological replicates. Different letters indicate significant differences (*p* < 0.05), according to Duncan’s new multiple range test.

We previously demonstrated that 1.0 mM NaHS markedly increased endogenous H_2_O_2_ accumulation, and H_2_S-induced H_2_O_2_ plays an important role in CO_2_ assimilation and photoprotection in cucumber [14,17]. Consistent with previous results, we found that NaHS induced endogenous H_2_O_2_ production. However, both N, N′-dimethylthiourea (DMTU, a H_2_O_2_ scavenger) and diphenyleneiodonium chloride (DPI, a H_2_O_2_ synthesis inhibitor) markedly inhibited the H_2_S-induced increase in H_2_O_2_ biosynthesis and Respiratory burst oxidase homolog (*RBOH1*) mRNA abundance in seedlings under chilling stress (Figure 2a,b). NaHS obviously decreased the CI and EL and increased *F*_v_/*F*_m_ and Φ_PSII_, but the NaHS-induced decrease in CI and EL or increase in *F*_v_/*F*_m_ and Φ_PSII_ in stressed seedlings were weakened by DMTU and DPI (Figure 2c–e). Therefore, we speculate that H_2_O_2_ is involved in the H_2_S-induced response to chilling stress.

### 2.2. H_2_O_2_ Participates in IAA-Induced Chilling Tolerance in Cucumber

**Figure 3:**
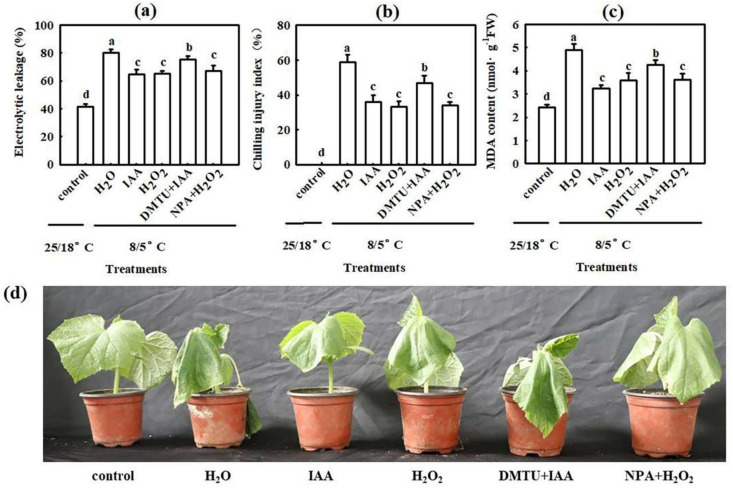
Interactive effects of IAA and H_2_O_2_ on the chilling tolerance of cucumber seedlings. Cucumber seedlings were pretreated with 75 μM IAA, 1.0 mM H_2_O_2_, 5.0 mM DMTU + 75 μM IAA, 50 μM NPA +1.0 mM H_2_O_2_, or deionized water (control) for 24 h and subsequently were exposed to chilling (8/5 °C, day/night). (**a**) EL of seedlings before (control) and after chilling stress for 48 h. (**b**) CI of seedlings before (control) and after chilling stress for 72 h. (**c**) MDA content of seedlings before (control) and after chilling stress for 48 h; (**d**) Phenotype characterization of different treatments before (control) and after chilling stress for 48 h. Deionized water-treated seedlings before chilling stress were used as the control. The experiments were repeated three times with similar results. A typical picture is shown here. The data represent the mean ± SD of three biological replicates. Different letters indicate significant differences (*p* < 0.05), according to Duncan’s new multiple range test.

Our previous study demonstrated that IAA acts as a downstream signaling molecule and is involved in H_2_S-induced chilling tolerance in cucumber seedlings [4]. To explore the interactions of H_2_S, IAA, and H_2_O_2_ in response to chilling stress, we studied the interaction between H_2_O_2_ and IAA in the chilling stress response in cucumber. We first measured the EL, CI, and malondialdehyde (MDA) content in cucumber seedlings pretreated with 75 μM IAA, 1.0 mM H_2_O_2_, 5.0 mM DMTU + 75 μM IAA, 50 μM 1-naphthylphthalamic acid (NPA, a polar transport inhibitor of IAA) + 1.0 mM H_2_O_2_, or deionized water, after exposure to 8/5 °C for 48–72 h. Seedlings pretreated with IAA and H_2_O_2_ showed remarkably lower EL, CI, and MDA content than H_2_O-pretreated seedlings during chilling stress (Figure 3a–c). The decrease in EL, CI, and MDA content in IAA treatment was blocked by DMTU, but the values in H_2_O_2_ pretreated seedlings were not significantly affected by the IAA polar transport inhibitor NPA. The IAA- and H_2_O_2_ pretreated seedlings exhibited distinctly less damage caused by chilling. The effects of IAA in mitigating in chilling damage in cucumber seedlings was weakened by DMTU, but NPA had minimal effect on H_2_O_2_-induced remission of chilling damage (Figure 3d).

**Figure 4:**
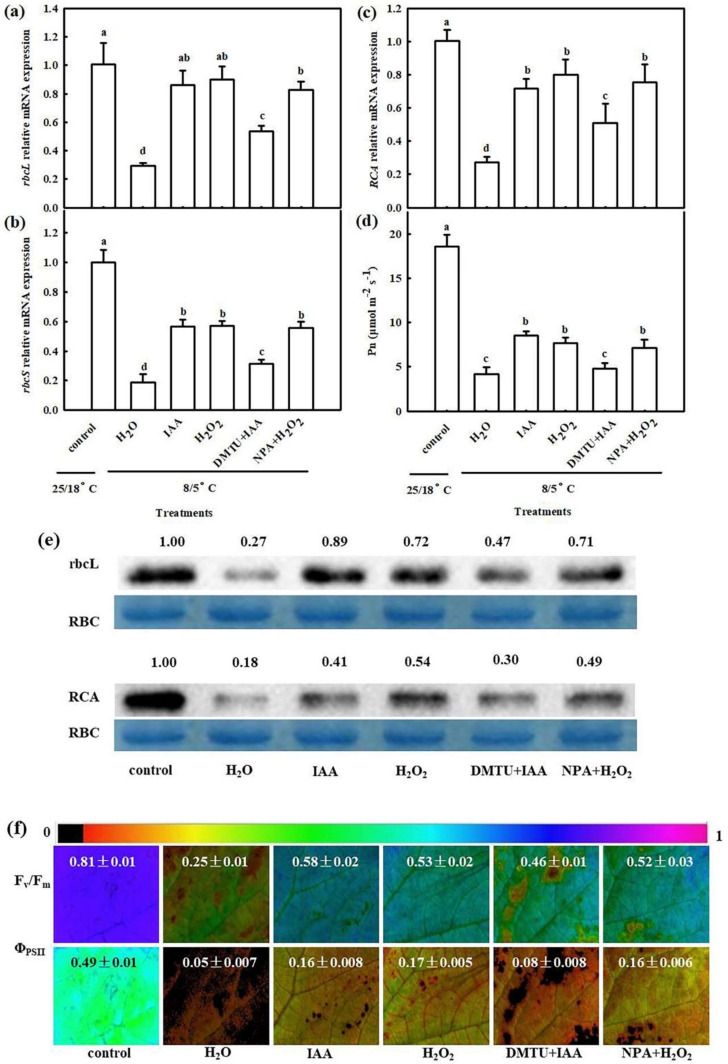
Interactive effects of IAA and H_2_O_2_ on mRNA abundances of *rbsL*, *rbcS,* and *RCA*, and protein levels of rbsL and RCA in cucumber seedlings under chilling stress. (**a**–**c**) mRNA abundances of *rbcL*, *rbcS,* and *RCA*; (**d**) Pn; (**e**) Protein levels of rbcL and RCA; (**f**) Image of *F*_v_/*F*_m_ and Φ_PSII_. The false color code depicted at top of the image represents the degree of photoinhibition at PSII. Cucumber seedlings were pretreated with 75 μM IAA, 1.0 mM H_2_O_2_, 5.0 mM DMTU +75 μM IAA, 50 μM NPA +1.0 mM H_2_O_2_, or deionized water (control) for 24 h, and subsequently exposed to 5 °C for 24 h. The data represent the mean ± SD of three biological replicates. Different letters indicate significant differences (*p* < 0.05), according to Duncan’s new multiple range test.

Then, we detected the interactive effects of IAA and H_2_O_2_ on the mRNA levels of large and small subunits *(rbcL*, *rbcS*) of ribulose 1, 5-bisphosphate carboxylase/oxygenase (rubisco) and rubisco activase (*RCA*), as well as the P_n_, *F*_v_/*F*_m_ and *Φ*_PSII_ under chilling stress. Both IAA and H_2_O_2_ treatments revealed a marked increase in mRNA levels of *rbcL*, *rbcS,* and *RCA* (Figure 4a–c), and rbcL and RCA protein levels (Figure 4e), compared with H_2_O treatment (*p* < 0.05). The application of DMTU distinctly repressed IAA-induced expression of *rbcL*, *rbcS,* and *RCA*, but NPA did not inhibit the effect of H_2_O_2_ on *rbcL*, *rbcS,* and *RCA* mRNA levels. Chilling stress significantly reduced the *P*_n_ of cucumber seedlings. After chilling treatment for 24 h, the decrease in P_n_ in cucumber seedlings was 77.6%, 53.9%, 58.6%, 74.2%, and 61.8% in the H_2_O, IAA, H_2_O_2_, DMTU + IAA, and NPA+ H_2_O_2_ treatments respectively, compared to the control (Figure 4d). Figure 4f shows that *F*_v_/*F*_m_ and *Φ*_PSII_ were markedly higher in IAA- and H_2_O_2_-treated than in H_2_O-treated seedlings during chilling stress. The application of DMTU significantly weakened the IAA-induced increase in *F*_v_/*F*_m_ and *Φ*_PSII_, but NPA showed a minimal influence on the H_2_O_2_-induced variation of *F*_v_/*F*_m_ and *Φ*_PSII_. These data suggest that IAA and H_2_O_2_ mitigate the negative effect of chilling stress on the photosynthetic function by upregulating the mRNA and protein levels of the key photosynthetic enzymes and activating the photoprotective mechanism.

**Figure 5:**
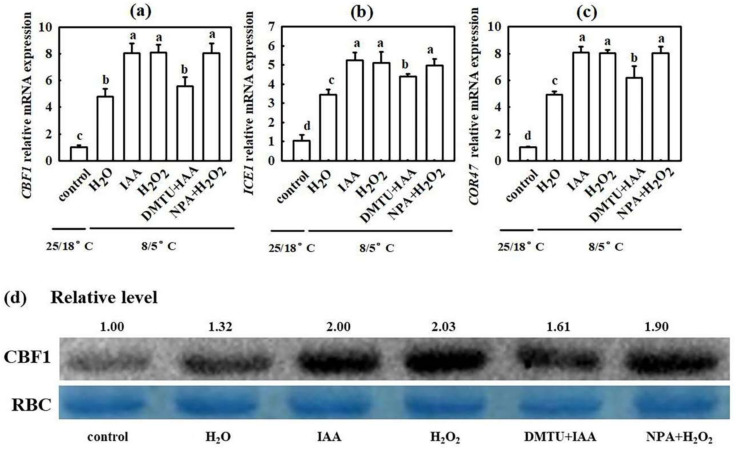
Interactive effects of IAA and H_2_O_2_ on the level of cold responsive genes in cucumber seedlings under chilling stress. (**a**–**c**) mRNA abundances of *CBF1*, *ICE1,* and *COR47,* respectively; (**d**) CBF1 protein level. Cucumber seedlings were pretreated with 75 μM IAA, 1.0 mM H_2_O_2_, 5.0 mM DMTU +75 μM IAA, 50 μM NPA +1.0 mM H_2_O_2_, or deionized water (control) for 24 h and subsequently exposed to 5 °C for 24 h. The data represent the mean ± SD of three biological replicates. Different letters indicate significant differences (*p <* 0.05), according to Duncan’s new multiple range test.

We also analyzed the effect of IAA and H_2_O_2_ on the relative expression of the cold responsive genes after seedlings were exposed to chilling stress for 24 h. IAA and H_2_O_2_ notably increased the mRNA levels of C-repeat-binding factor (*CBF1*), inducer of *CBF* expression (*ICE1*) and cold responsive (*COR47*) genes (Figure 5a–c) as well as CBF1 protein levels (Figure 5d) in cucumber seedlings under chilling stress. The increases in the mRNA and protein levels of the cold responsive genes in IAA-treated seedlings were dramatically weakened by DMTU, whereas those in H_2_O_2_-treated seedlings were minimally affected by NPA.

**Figure 6:**
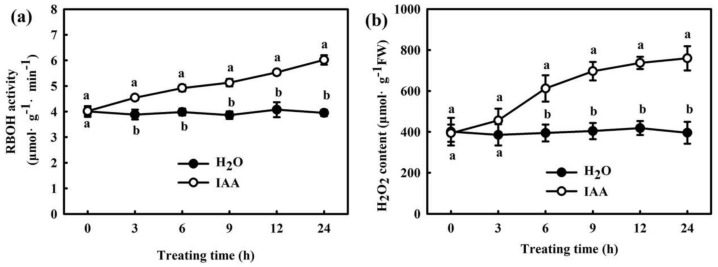
Effect of IAA on the RBOH activity (**a**) and H_2_O_2_ accumulation (**b**) in cucumber seedlings. Cucumber seedlings were foliar sprayed with 75 μM IAA or deionized water (control), and then, we measured the changes of RBOH activity and H_2_O_2_ content within 24 h. The data represent the mean ± SD of three biological replicates. Different letters indicate significant differences (*p* < 0.05), according to Duncan’s new multiple range test.

**Figure 7:**
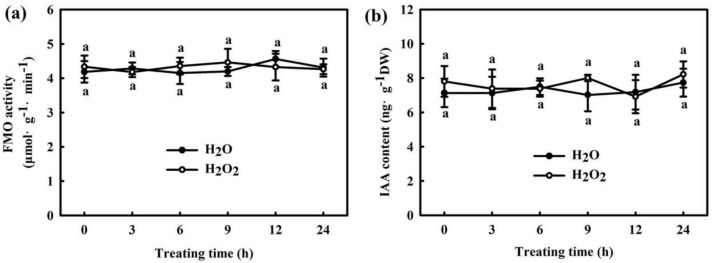
Effects of H_2_O_2_ on the FMO activity (**a**) and IAA content (**b**) in cucumber seedlings. Cucumber seedlings were foliar sprayed with 1.0 mM H_2_O_2_ or deionized water (control), and then, we measured the changes of FMO activity and IAA content within 24 h. The data represent the mean ± SD of three biological replicates. Different letters indicate significant differences (*p* < 0.05), according to Duncan’s new multiple range test.

We found that 75 μM IAA remarkably increased the RBOH activity (Figure 6a) and H_2_O_2_ content (Figure 6b) in cucumber seedlings, and the increase was remarkable after treatment for 6 h. However, no remarkable differences in flavin monooxygenase (FMO) activity and IAA content were observed between H_2_O_2_- and H_2_O-treated seedlings (Figure 7). At normal temperature, the H_2_O_2_-treated seedlings showed similar mRNA expressions of *PIN1* and *AUX2* to the H_2_O-treated seedlings. After 9 h or 24 h of chilling stress, *PIN1* and *AUX2* mRNA levels markedly increased in both H_2_O_2_ and H_2_O treatment, but the extent of the increase did not vary and showed no significant differences between H_2_O_2_ and H_2_O_-_treated seedlings (Supplemental Figure S1). All the above results indicate that IAA affects H_2_O_2_ signaling in cucumber seedlings under chilling stress. H_2_O_2_ might play a critical role in the IAA-induced positive response to chilling stress in cucumber seedlings.

### 2.3. Interaction of IAA and H_2_O_2_ in H_2_S-Induced Chilling Tolerance in Cucumber

**Figure 8:**
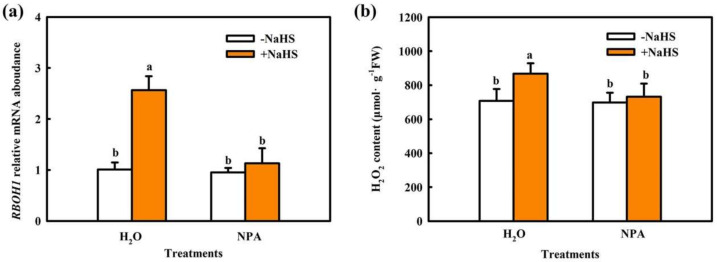
Effect of NPA on H_2_S-induced *RBOH1* mRNA abundance (**a**) and H_2_O_2_ accumulation (**b**) in cucumber seedlings. Cucumber seedlings were pretreated with 50 μM NPA or deionized water and then sprayed with 1.0 mM NaHS after 6 h. Twelve hours later, the seedlings were exposed to 5 °C for 9 h. The data represent the mean ± SD of three biological replicates. Different letters indicate significant differences (*p* < 0.05), according to Duncan’s new multiple range test.

**Figure 9:**
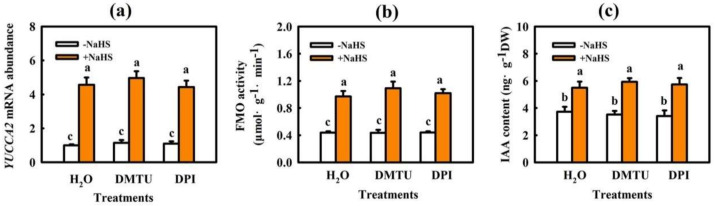
Effect of DMTU and DPI on H_2_S-induced IAA production in cucumber seedlings. (**a**) *YUCCA2* mRNA abundance; (**b**) FMO activity; (**c**) IAA accumulation. Cucumber seedlings were pretreated with 5.0 mM DMTU, 100 μM DPI, or deionized water and then sprayed with NaHS after 6 h. Twelve hours later, the seedlings were exposed to 5 °C for 9 h. The data represent the mean ± SD of three biological replicates. Different letters indicate significant differences (*p <* 0.05), according to Duncan’s new multiple range test.

To further analyze the upstream and downstream relationship between IAA and H_2_O_2_ in H_2_S-mediated plant stress response, we determined the effect of NPA on H_2_S-induced H_2_O_2_ production and that of the H_2_O_2_ scavenger DMTU and H_2_O_2_ synthetic inhibitor DPI on H_2_S-induced IAA biosynthesis. As shown in Figure 8, 1.0 mM NaHS markedly increased *RBOH1* mRNA abundance and H_2_O_2_ accumulation. NPA significantly inhibited the increase in *RBOH1* mRNA abundance and H_2_O_2_ content induced by NaHS, suggesting that IAA is involved in H_2_S-induced H_2_O_2_ production. NaHS also upregulated FMO-like protein *(YUCCA2)* mRNA abundance, FMO activity, and IAA levels, but DMTU and DPI had minimal effects on H_2_S-induced IAA biosynthesis in cucumber leaves (Figure 9). Combining the results of Figure 2, it is further inferred that H_2_O_2_, a downstream component of IAA, is involved in H_2_S-induced chilling tolerance in cucumber seedlings.

## 3. Discussion

Chilling is a major abiotic stress that affects the growth, development, and geographical distribution of plants [18,19]. Low-temperature stress mainly affects light energy utilization and photosynthetic efficiency by destroying electron transport chains in chloroplasts and mitochondria, leading to ROS accumulation, and eventually inducing cell membrane damage in plants [20]. H_2_S, as a major gaseous transmitter, plays a critical role in plant resistance to various stress conditions, such as low temperature, salt, drought, and heavy metals [21,22]. The application of exogenous H_2_S can enhance chilling tolerance in *Arabidopsis thaliana* [1], hawthorns [23], and cucumbers [3]. ABA, NO, Ca^2+^, and SA are involved in H_2_S-induced resistance to abiotic stresses in plants [24,25,26]. Recently, we verified that H_2_S interacts with NO, ABA, Ca^2+^, IAA, and SA to enhance the chilling tolerance in cucumbers [4,5,6,7,27]. However, whether H_2_O_2_ and IAA exhibit synergistic effects on the H_2_S-mediated plant stress response remains unclear.

Previous studies have revealed that H_2_O_2_ is a key molecule of signal transduction and regulates various physiological metabolic processes. For example, H_2_O_2_ recruited the promoter of the senescence-related transcription factor WRKY53, which in turn activated WRKY53 transcription and led to a senescence of *Arabidopsis* [28]. The H_2_O_2_ response gene (*HRG1/2*) could quickly respond to exogenous or endogenous H_2_O_2_ and further regulated *Arabidopsis* seed germination [29]. Islam et al. proved that by inducing production of the reactive carbonyl species (RCS), H_2_O_2_ could induce stomatal closure of guard cells in *Arabidopsis* [30]. Moreover, H_2_O_2_ responds to many abiotic stress of plants. Sun et al. showed that as a signal, on the one hand, Respiratory burst oxidase homologue-dependent H_2_O_2_ (RBOH-H_2_O_2_) enhanced the heat tolerance of heat sensitive tomato. On the other hand, RBOH-H_2_O_2_ regulated the activities of antioxidant enzymes to control the total H_2_O_2_ at a level conducive to heat stress memory, which in turn maintained a lower level of total H_2_O_2_ during the future heat stress challenge [8]. H_2_O_2_ also could interact synergistically with other hormones or regulators, such as IAA, ABA, SA, MeJA, etc., mediating the response to abiotic stress in plants [10,31]. Recently, our results showed that H_2_O_2_ induced CO_2_ assimilation and photoprotection in cucumber seedlings during chilling stress [14]. In this study, we found that H_2_O_2_ increased the chilling tolerance of cucumber seedlings (Figure 1), suggesting that the response of H_2_O_2_ to chilling stress is consistent with previous studies. NaHS significantly enhanced H_2_O_2_ levels, *RBOH1* mRNA abundance, and chilling tolerance in cucumber seedlings. The H_2_O_2_ scavenger DMTU and inhibitor of H_2_O_2_ synthesis DPI decreased H_2_S-induced H_2_O_2_ accumulation and chilling tolerance (Figure 2). These results indicate that H_2_O_2_ may crosstalk with H_2_S to improve the chilling stress response in cucumber seedlings.

Auxin is a major phytohormone that controls various aspects of plant growth and development, including cell division and elongation, tissue patterning, and the response to environmental stimuli [32,33], but knowledge about its role and interaction with other signals under chilling stress is limited. Previous investigations have indicated that chilling-induced variation in plant growth and development is closely related to the intracellular auxin gradient. Chilling stress promotes auxin biosynthesis or changes auxin gradient distribution, thus affecting the root gravity response in Arabidopsis, rice, and poplar [15,34,35,36]. Recently, we learned that NaHS increased endogenous IAA accumulation and improved chilling tolerance. The IAA polar transport inhibitor NPA suppressed H_2_S-induced chilling tolerance. IAA reduced the negative effects of chilling stress on growth and photosynthesis, but it showed minimal effects on endogenous H_2_S levels. H_2_S scavengers did not influence the chilling tolerance induced by IAA [4]. Here, we observed that IAA-induced chilling tolerance was repressed by the H_2_O_2_ scavenger DMTU, but the IAA inhibitor NPA did not affect H_2_O_2_–induced tolerance to chilling stress (Figure 3, Figure 4 and Figure 5). IAA significantly enhanced endogenous H_2_O_2_ synthesis, but H_2_O_2_ showed minimal effects on endogenous IAA level in cucumber seedlings (Figure 6 and Figure 7). Thus, we speculate that IAA depends on the H_2_O_2_ signaling pathway in the regulation to chilling stress response. In addition, NPA significantly decreased H_2_S-induced *RBOH1* and H_2_O_2_ levels (Figure 8), whereas DMTU and DPI showed no marked effect on H_2_S-induced *YUCCA2* mRNA abundance, FMO activity, or IAA levels (Figure 9). These results suggest that H_2_O_2_ lies downstream of IAA in the regulation of H_2_S to the chilling stress response.

**Figure 10:**
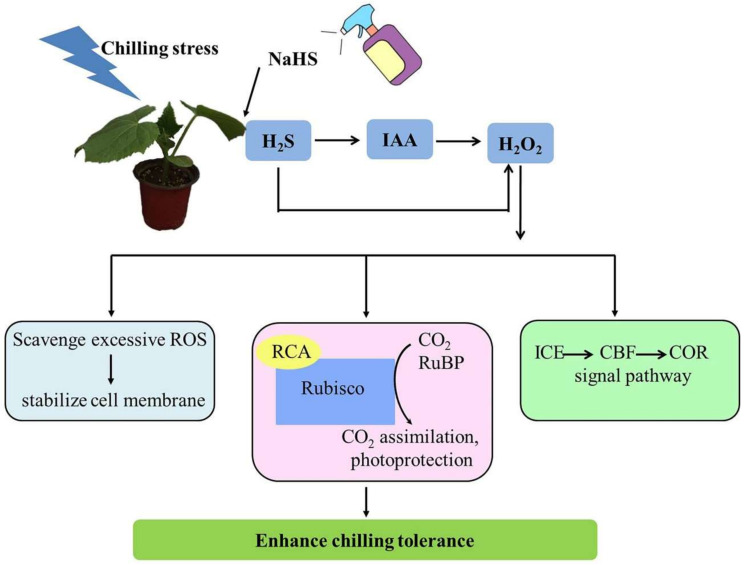
A proposed model for the role of IAA and H_2_O_2_ in H_2_S-induced chilling tolerance in cucumber. Chilling induces the accumulation of H_2_S in the plants. H_2_S induced by chilling promotes IAA generation, triggers H_2_O_2_ accumulation, and subsequently increases chilling tolerance by scavenging excessive ROS, improving CO_2_ assimilation and photoprotection, and upregulating the levels of cold-responsive genes.

Based on previous studies and the above results, we proposed a model of H_2_O_2_ and IAA regulating the H_2_S-mediated chilling stress response in cucumber seedlings. Figure 10 shows that endogenous H_2_S induced by chilling stress or the application of exogenous NaHS, H_2_O_2_, and IAA all enhanced chilling tolerance in cucumber seedlings by scavenging excessive ROS, improving photosynthetic capacity, and upregulating the mRNA and protein levels of cold responsive genes. Chilling stress-induced or exogenous H_2_S promotes IAA generation, and IAA further triggers H_2_O_2_ accumulation and subsequently increases chilling tolerance. Thus, H_2_O_2_ may act as a downstream signal of IAA and play a significant role in H_2_S-mediated chilling stress tolerance in cucumber seedlings. Further studies using advanced molecular techniques and mutants are required to better reveal the mechanisms and interactions of H_2_S-, IAA-, and H_2_O_2_-induced chilling tolerance in plants.

In summary, H_2_O_2_ and IAA markedly improved the chilling tolerance of cucumber seedlings, as illustrated by the decrease in stress-induced CI and EL, the increase in CO_2_ assimilation, and the upregulation in the level of cold-responsive genes. Even more importantly, our results first confirmed that H_2_O_2_ interacts with IAA signaling and is jointly involved in H_2_S-induced chilling tolerance in cucumber. Moreover, H_2_O_2_ may act as a downstream signaling molecule of IAA, and it plays a critical role in H_2_S-mediated chilling stress response in cucumber.

## 4. Materials and Methods

### 4.1. Plant Materials and Growth Condition

“Jinyou 35” cucumber (*Cucumis sativus* L.) seedlings were used in the current study. After soaking and germinating, the seeds were sown in nutrition bowls filled with seedling substrate, which consisted of peat, vermiculite, and perlite (5:3:1, *v*/*v*), and then transferred to a climate chamber with a photon flux density (PFD) of 600 μmol m^−2^·s^−1^, a 26/17 °C thermoperiod, an 11 h photoperiod, and 80% relative humidity.

### 4.2. Experimental Design

#### 4.2.1. Effect of H_2_O_2_ on the Chilling Tolerance of Cucumber Seedlings

The seedlings with two leaves were foliar sprayed with 0 (control), 0.01, 0.1, 1.0, 2.0, and 3.0 mM H_2_O_2_, respectively. Twenty-four hours later, the pretreated seedlings were exposed to 8/5 °C to analyze the CI, EL, P_n_, F_v_/F_m_, and Φ_PSII_.

#### 4.2.2. Effect of H_2_O_2_ Scavenger or Inhibitor on H_2_S-Induced H_2_O_2_ Biosynthesis and Chilling Tolerance

The seedlings were pretreated with 1.0 mM NaHS, (a H_2_S donor), 5.0 mM DMTU (a H_2_O_2_ scavenger), 100 μM DPI (a H_2_O_2_ synthesis inhibitor), 5.0 mM DMTU + 1.0 mM NaHS, 100 μM DPI + 1.0 mM NaHS, or deionized water (H_2_O). Twenty-four hours later, the pretreated seedlings were subjected to 8/5 °C for 9–72 h to assay the biosynthesis of H_2_O_2_, EL, CI, F_v_/F_m_, Φ_PSII_ and relative expression of cold-responsive genes. The H_2_O treatment at normal temperature served as the control.

#### 4.2.3. Interaction between IAA and H_2_O_2_ in Response to Chilling Stress

The seedlings were pretreated with 75 μM IAA, 1.0 mM H_2_O_2_, 5.0 mM DMTU + 75 μM IAA, 50 μM NPA (a polar transport inhibitor of IAA) +1.0 mM H_2_O_2_, or deionized water (H_2_O). At 24 h after pretreatment, the seedlings were exposed to 8/5 °C to assay the P_n_, fluorescence parameters, gene expression and protein expression of key photosynthesis enzymes, and relative expression of cold-responsive genes. H_2_O treatment under normal conditions served as the control.

#### 4.2.4. Effect of IAA Inhibitor on H_2_S-Induced H_2_O_2_ Biosynthesis in Cucumber Seedlings

The seedlings were pretreated with 1.0 mM NaHS, 50 μM NPA, 50 μM NPA + 1.0 mM NaHS, or deionized water (H_2_O). Twenty-four hours later, the pretreated seedlings were subjected to 5 °C for 9 h to assay the relative expression of *RBOH1* and H_2_O_2_ content.

#### 4.2.5. Effect of Scavengers and Synthetic Inhibitors of H_2_O_2_ on H_2_S-Induced IAA Biosynthesis in Cucumber Seedlings

The seedlings were pretreated with 1.0 mM NaHS, 5.0 mM DMTU, 100 μM DPI, 5.0 mM DMTU + 1.0 mM NaHS, 100 μM DPI + 1.0 mM NaHS, or deionized water (H_2_O). Twenty-four hours later, the pretreated seedlings were subjected to 5 °C for 9 h to assay the FMO activity, *YUCCA2* mRNA abundance, and IAA content.

### 4.3. CI, EL, and MDA Measurements

The chilling stressed cucumber seedlings were graded based on the Semeniuk et al. [37] standard, and the CI was calculated according to the following formulas: CI = Σ (plants of different grade × grade)/[total plants × 5 (the maximum grade)].

EL was measured as described by Dong et al. [38]. Leaf discs (0.2 g) were incubated at 25 °C in 20 mL deionized water for 3 h, and the electrical conductivity (EC1) was estimated using a conductivity meter (DDB-303A, Shanghai, China). Afterwards, the leaf discs were boiled for 30 min and then cooled to detect EC2. EL was calculated according to the following formula: EL= EC1/EC2 × 100.

MDA content was determined using the thiobarbituric acid (TBA) colorimetric method as described by Heath and Packer [39].

### 4.4. Detection of P_n_ and Chlorophyll Fluorescence 

The P_n_ was determined using a portable photosynthetic system (Ciras-3, PP-systems International, Hitchin, Hertfordshire, UK). Constant PFD (600 μmol·m^−2^·s^−1^), CO_2_ concentration (380 mg·L^−1^), and leaf temperature (25 ± 1 °C) were maintained during the assessment. *F_v_/F_m_* was measured after seedlings were dark-adapted for 45 min, and the Φ_PSII_ was determined after leaves were light-adapted for 30 min using a portable pulse-modulated fluorometer (FMS-2, Hansatech, King’s Lynn, Norfolk, UK). The chlorophyll fluorescence parameters were calculated according to Demmig-Adams and Adams [40] and Maxwell [41] as follows: F_v_/F_m_ = (F_m_−F_0_)/F_m_′; Φ_PSII_ = (F_m_′−F_s_)/F_m_′. Chlorophyll fluorescence imaging was visualized using a chlorophyll fluorescence imaging system (Imaging PAM, Walz, Wurzburg, Germany) with a computer-operated PAM-control system [42].

### 4.5. Detection of H_2_O_2_ Content and RBOH Activity

H_2_O_2_ content was determined using an H_2_O_2_ kit (Nanjing Jiancheng Bioengineering Institute, Nanjing, China). RBOH activity was detected with an ELISA kit (Jiangsu Meimian Industrial Co. Ltd., Yancheng, China) according to the instructions.

### 4.6. IAA Content and FMO Activity Assay

IAA content was determined by the method of Zhang et al. [4]. In brief, 0.3 g sample ground with liquid nitrogen and extracted thrice with 80% methanol (containing 30 μg·mL^−1^ sodium diethyldithiocarbamate). Samples were centrifuged (7155 g, 10 min, 4 °C) to obtain the supernatant by rotary evaporation (Shanghai EYELA, N-1210B, Shanghai, China) at 38 °C. The residue was washed with 5 ml of PBS (pH = 8, 0.2 M) and 4 mL of trichloromethane, shaken for 20 min, and allowed to stand for 30 min to remove pigment present in the trichloromethane. The resulting residues were added to 0.15 g of polyvinylpolypyrrolidone (PVPP) to remove phenols. Then, the samples were centrifuged at 7155 g for 10 min, and the resulting supernatant was re-extracted with ethyl acetate thrice and dried with a rotary evaporator in vacuo at 36 °C. The dried material was dissolved in 1.0 mL mobile phases (methanol: 0.04% acetic acid = 45:55, *v*/*v*), and the filtrate was used for HPLC–MS (Thermo Fisher Scientific, TSQ Quantum Access, San Jose, CA, USA) analysis followed by the method of Zhang et al. [4].

Flavin monooxygenase (FMO) activity was estimated using an ELISA kit (Jiangsu Meimian Industrial Co. Ltd., Yancheng, China). In brief, the FMO1 antibody was conjugated with standard, sample, and horseradish peroxidase (HRP)-labeled detection antibody and incubated, aspirated, and washed. Then, chromogen solution was added, and the reaction was terminated with sulfuric acid solution. The absorbance was detected at 450 nm with a microplate reader, and FMO activity was calculated using the standard curve [4].

### 4.7. Quantitative Real-Time PCR Analysis

Total RNA was extracted from cucumber leaves using an RNA extraction kit (TRIzol; Tiangen, Beijing, China). The isolated RNA was reverse transcribed with the PrimeScript^®^ RT Master Mix Perfect Real Time (TaKaRa, Dalian, China). qRT-PCR was performed using the TransStart^®^ TipTop Green q-PCR SuperMix (Cwbio, Beijing, China). The relative expression levels were standardized to those of cucumber β-actin gene (Solyc11g005330). The qRT-PCR primers are shown in Supplemental Table S1.

### 4.8. SDS-PAGE and Immunoblot Analysis

The extracted total protein of samples was separated using a 10% SDS-PAGE gel, and the resulting proteins were transferred to polyvinylidene difluoride (PVDF) membranes. The PVDF membrane was blocked for 2 h with 5% (*w*/*w*) skimmed milk and then incubated with the primary antibody followed by a horseradish peroxidase-conjugated anti-rabbit IgG antibody (ComWin Biotech Co., Ltd., Beijing, China) for 2 h. Finally, the immunoreaction was tested using the eECL Western blot Kit (CW00495, ComWin Biotech Co., Ltd., Beijing, China) and the ChemiDoc™ XRS imaging system (Bio-Rad Laboratories, Inc., Hercules, CA, USA). Primary antibodies against RbcL and RCA (ATCG00490, AT2G39730) were obtained from PhytoAB Co. Ltd. (San Francisco, CA, USA), and the CBF1 antibody was obtained from GenScript Co., Ltd. (Nanjing, China).

### 4.9. Statistical Analysis

The whole experiment was performed in triplicate, and the results shown are the mean ± standard deviation (SD). Statistical analysis was performed with DPS software, and the comparison of treatments was based on the analysis of variance by Duncan’s multiple range test (DMRT) at a significance level of 5% (*p* < 0.05).

## Data Availability

The original contributions presented in the study are included in the article, further inquiries can be directed to the corresponding author.

## Supplementary Materials

The following are available online at https://www.mdpi.com/article/10.3390/ijms222312910/s1.
