# Supplementary material for: H2O2 Functions as a Downstream Signal of IAA to Mediate H2S-Induced Chilling Tolerance in Cucumber
Source: Int J Mol Sci. 2021 Nov 29;22(23):12910. doi: 10.3390/ijms222312910 (PMC8657662; doi:10.3390/ijms222312910)
Supplement: Supplementary file 1 [file ijms-22-12910-s001.zip › ijms-1438283-supplementary.pdf]

International Journal of *Molecular Sciences*

MDPI

H_2O_2 Functions as a Downstream Signal of IAA to Mediate H_2S-Induced Chilling Tolerance in Cucumber

Xiaowei Zhang, Yanyan Zhang, Chenxiao Xu, Kun Liu, Huangai Bi and Xizhen Ai *

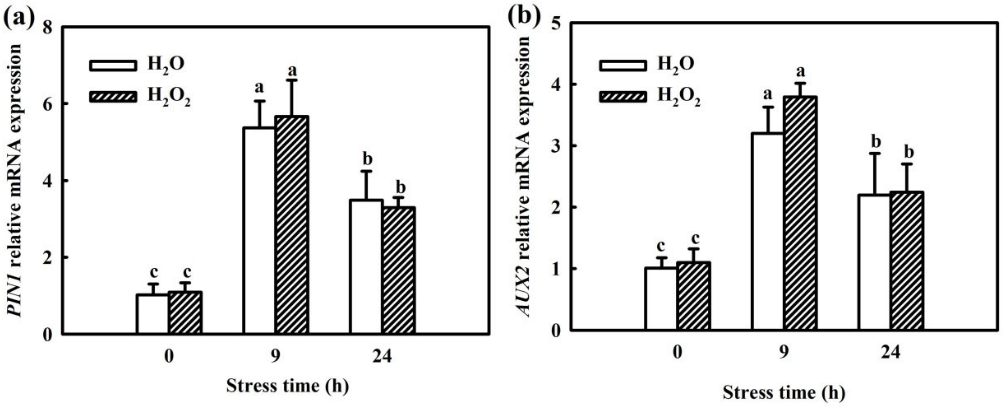

Figure S1. Effect of H_2O_2 on the mRNA expressions of *PIN1* and *AUX2* under chilling stress. (a) qRT-PCR analysis the expression of *PIN1*; (b) qRT-PCR analysis the expression of *AUX2*. Cucumber seedlings were pretreated with 1.0 mM H_2O_2 or deionized water (H_2O) for 6 h, and subsequently were exposed to chilling stress (5 °C) for 24 h. qRT-PCR was performed in three biological replicates and three technical replicates. Deionized water treated seedlings before chilling stress were used as the control. Error bars denote standard deviations. Different letters above the bars indicate significant differences ($P < 0.05$) based on Duncan's multiple range tests.

Table S1. Primers for qRT-PCR analysis.

Gene	Sequences
RBOH1	F: TTGCTGGGAAGAGTGGGT
	R: GCTCCAATACCAAGACCAAC
YUCCA2	F: TTCTGTCAACTTCCCAACTTCCCTT
	R: GGAAGTTGGGAAGTTGACAGAATTG
RCA	F: AAAGTGGGCTGTAGGCGTTG
	R:CTTTTCTATTGTCATCTTCGGTTGG
rbcL	F:GCTATGGAATCGAGCCTGTTG
	R:CCAAATACATTACCCACAATGGAAG
rbcS	F:CGCATTCATCAGGGTTATTGG
	R:AAGAGTAGAACTTGGGGCTTGTAGG
CBF1	F:ATGGCTTCATATTGCTCTGAG
	R:ATGCCTCAAGTCAATTGCTTG
ICE1	F:CGCATCGAGTTGGCTCTGGTG

	R:GTCCTCATCGCCGTTCATCTTCC
COR47	F:CACTTTGAGAGGACATTTGATG<br>R:AGAAGCTCCAATTTTGACTTG
PIN1	F: GTAGAGAGAACCAGGAGGAA<br>R: CAAGATTAGCCTTGTCATAA
AUX2	F: CTTCTAAACCATTCCAACGC<br>R: AAACAAATGCTCTTCGTATC
Actin	F:CCACGAAACTACTTACAACTCCATC<br>R:GGGCTGTGATTTCCTTGCTC
